# Supplementary material for: Economic analysis of avocado production systems: Market failures and policy distortions
Source: PLoS One. 2025 Aug 25;20(8):e0330326. doi: 10.1371/journal.pone.0330326 (PMC12377620; doi:10.1371/journal.pone.0330326)
Supplement: Attachment — Submitted filename: Comment_SSM.pdf [file pone.0330326.s004.pdf]

Line 131, “with ranges” should be replaced with “with age ranges”.

Line 280, you mentioned in Table 2 for APU2 (7-9), APU3 (10 to 14), in Table 3 for APU3 (7m10a14) and in Table 5 for APU2 (6-9), APU3 (>10), with respective age ranges, but they are not in line with what in materials and methods (Line 132-133, where APU1 (4-6 years), APU2 (8-9 years), APU3 (10-15 years). Their descriptions should be consistent throughout the paper.

In Table 2 (Line 280), there is a misspelling, “tres”, which should be “trees”.

The names of Table 1, 2, and 3 are too short and not self-explained, which need a bit more words.

Figure 2 does not show any unit.

Line 498, 542, the quotes (“”) are not necessary for the words “harms” and “distorting”.

You referred Table 6 in Lines, 341,354,459,467,375, but I could not find the Table 6 anywhere.

In Table 4, you need the unit (m or meter) in column 2.

In Figure 3, the APUs is described in title, but it was not included in figure, instead you mentioned 6m4a6, 6m7a9 and 7m10a14, for which I would suggest just to write APU1, APU2 and APU3 simply.
