# Supplementary material for: Economic analysis of avocado production systems: Market failures and policy distortions
Source: PLoS One. 2025 Aug 25;20(8):e0330326. doi: 10.1371/journal.pone.0330326 (PMC12377620; doi:10.1371/journal.pone.0330326)
Supplement: Attachment — Submitted filename: Rebuttal letter.docx [file pone.0330326.s002.docx]

In response to the reviewers' feedback, the following revisions were made to the original manuscript:

**Reviewer 1:**

| Introduction: Line 33 Don't you have a more up-to-date figure? | New paragraphs were included:  **Lines 32-37** in the revised manuscript: “*Mexico leads… …3, 4]*”  **Lines 38-45**: “*The avocado… … [6]*”.  to reflect updated data. |
| --- | --- |
| The topic should be delved into if there are studies related to the cultivation of avocado, at the Mexico and state level, or related studies to see the methodological viability in important crops, in Michoacán or Jalisco. | New paragraphs were added to the introduction to address avocado studies in Mexico and demonstrate the methodology's relevance to key crops at both national and global levels.  **Lines 46-52:** “*In recent… …[8].*”  **Lines 53-57:** “*In Mexico,… …[9].*”  **Lines 58-63:** “*The State of Mexico… …[3].*”  **Lines 64-74:** “*Comparative… ….resource allocation.*”  **Lines 75-77:** “*The policy… …[16, 17].*”  **Lines 78-84:** “*This methodology… …Malaysia.*”  The final paragraph of the introduction was refined for improved clarity.  **Lines 85-91:** “*This study… …region of Mexico.*” |
| Material and methods: Line 52 – 54 add a location map. Delve into the place of study, if it is important in the production of avocado, what ranking it occupies in the state, among other characteristics that describe the relevance of the crop in the place. | A properly cited map showing Ocuilan's relief and coordinates was included.  **Line 97:** “*Figure 1: Map … …. Mexico.*”  Ocuilan's temperature range and precipitation were included, along with its ranking in avocado production within the State of Mexico..  **Line 95-96**: “*The area… … agroecological zone.*” |
| Line 64 What was the density? | **Line 109:** “*120-366 trees per hectare*” |
| Line 115 – 116 Is the product produced in this area imported to the USA? How much? | It was clarified in the revised manuscript that exports to the U.S. do not occur.  **Lines 181-183:** “*Both analyzed… …[9].*” |
| It is understood that the avocado produced at this site is the same as that produced in Michoacán. The characteristics of avocados in both areas must be different. These differences must be clarified. On the political side, it is homogenized in the country. | New lines were added to specify that the analyzed avocado systems are small-scale. It is also noted that Mexico's support policies apply uniformly to all avocado systems.  **Lines 108:** “*All APUs were small… …technology.*”  **Lines 109-111:** “*The support… …climatic región.*” |
| Results: line 201 – 216 this part should go in methodology. | The paragraphs were moved to the methodology section, under *Modelling of production systems.* **Lines 124-141.** |
| Line 218-220 compare it with the production of Michoacán or Jalisco. | Literature was added to align with studies from Michoacán, Jalisco, and the State of Mexico.  **Lines 268-269:** “*These tree… …36].*” |
| Line 320 -322 What is mentioned is relevant. Since production is new in the municipality and wild avocado is used, are these studies relevant to this type of avocado? | The study focused on commercial plantations (non-subsistence) cultivating Hass avocados. Older plantations are 8–14 years old, while younger ones are 6 years old. Additional details were added to discuss the impact of the Hass variety on native avocados.  **Lines 370-372**: “*A clear trend… …[39].*” |
| Line 335 And to national yield? | The national yield was included.  **Line 386**: *“, while the national… …hectare.”* |
| Line 345-349 Is it the same setting as your studio? | It is a comparable study conducted in the State of Mexico, but in a different region. |
| Line 356-357 Go deeper into the topic | Additional lines were added to delve deeper into the topic.  **Lines 410-414**: “*In 2020,… …transfers.”* |
| Line 366 I don't think it's nationwide. | This point was clarified in the manuscript:  **Lines 109-111:** “*The support… …climatic región.*” |
| Line 387-389 national or foreign consumers? | It was clarified that taxes are borne by the domestic consumer.  **Line 446: “***the end national consumer…”* |
| Line 398-399 It is a confusing conclusion. Is the scenario presented in this study representative of the situation in Mexico? It is important to put similar studies done in the most productive avocado municipalities. | To avoid confusión the following lines were included:  **Lines 455-460**: “*Small-scale… …[17, 46].”* |
| Line 399 – 400 They are not developing countries, they are emerging countries. | Developing was changed to emerging. |
| Line 410-412 This part is worth highlighting in the manuscript, it presents 2 or more scenarios in the case of avocado production and marketing in Mexico. Which one is this? | The scenarios cover both production and marketing.  A paragraph was added to clarify this point.  **Lines 484-490**: “*An effect… …deprivation*”. |
| Line 431-433 This happens in many crops in Mexico. | This indeed occurs with many crops in Mexico. |
| Conclusions: go deeper into the conclusions | The conclusion of the study was expanded.  **Lines 513-536:** “*To assess… …this study.*” |
| The study is important; however, it presents a scenario that does not correspond to the Mexican avocado. It should be particularized only in the situation of the State of Edomex and in the municipality. What happens with production, marketing, export, etc., is more complicated than the scenario presented in this manuscript. | The study represents 2% of the avocado cultivation area in the State of Mexico but is considered representative of its production system. Consensus panel results reflect not only this 2% but also 44% of the state’s total avocado production. |

**Reviewer 2:**

| Line 33: the author referred to export information in 2020 during Covid-19. Such a year was the exception, so I suggest using export data for the time after that. Also, the data was collected during/right after the pandemic, so it is important to discuss the applicability of the results after that. | New paragraphs were added to include post-COVID literature, along with a discussion on the results' applicability.  **Lines 32-84:** “*Mexico leads… …Malaysia*.”  **Lines 560-562:** “*The authors… …consumption.*” |
| --- | --- |
| The introduction needs to expand more, detailing related policies within Mexico and providing information about international trade, such as the trade balance for avocados, and information about the importance of avocados, such as the ratio of planted areas or agricultural production or the contribution to the agricultural GDP. Also, it needs to detail relevant previous studies, identify the gap in the literature, and the contribution of this paper in filling this gap. | The introduction was revised to expand the literature and include updated figures. The final paragraph was rewritten to emphasize the significance of this study  Lines 32-84.  **Lines 85-91:** “*This study… …of Mexico.”* |
| Line 78: equation 1 is not clear; please explain more. | The equation was rewritten.  **Line 142** |
| Line 79: 79: what does “dose per lot” mean? Not clear. Also, a typo-delete one parenthesis. | “*dose per lot*” was changed to “*dose per surface*”.  **Line 143** |
| Line 132: I suggest starting the sentence with the “private profitability” phrase. | The observation has been addressed.  **Line 197** |
| Line 137: it says that “(J=B-F); transfers due to the price of internal factors.” This is inconsistent with table 1 indicating “(J=B-F) as transfer by commercial inputs, not internal factors. Please clarify. | There was indeed an error, which has been corrected in the text.  **Line 228** was removed: “*Transfers due to the price of internal factors*” |
| Line 236: not clear how APU2 is more profitable than APU1, while the average harvest is 44kg/ tree, which is lower than that of APU1. Please clarify. | The error was corrected: the average is 144 kg, not 44.  **Line 267.** |
| Lines 330: The studies by [7, 23, 25] reported… | The observation has been addressed.  **Line 380.** |
| typo within, not withing. | The observation has been addressed. |
| Line 387: it could also be that the avocado demand is inelastic, in which the tax is passed on to the consumers. | Our study focuses on evaluating the impact of policy or tax cascades on avocado producers, not elasticity. |
| Finally, I suggest discussing limitations, policy implications, and future research. | To clarify the conclusions, the hypothesis is addressed through two questions, each answered and supported with arguments. Proposals for future research are also included, considering the paper's limitations. |
