# Supplementary material for: Economic analysis of avocado production systems: Market failures and policy distortions
Source: PLoS One. 2025 Aug 25;20(8):e0330326. doi: 10.1371/journal.pone.0330326 (PMC12377620; doi:10.1371/journal.pone.0330326)
Supplement: Attachment — Submitted filename: PONE-D-24-45504R1 (Rebuttal letter) .docx [file pone.0330326.s003.docx]

In response to the reviewers' feedback, the following changes were made to the original manuscript:

**Reviewer 1:**

| **Introduction:** Line 33 Mexico is not a price setter. Line 49 – 50 the problems are stronger in states that want to excel in avocado exports. | Line 33 was removed, and a new paragraph was added to highlight the related issue.  Lines 48-54: “Avocado cultivation… … [4, 6].” |
| --- | --- |
| Line 53 – 54 phytosanitary programs are concentrated in the state that produces and exports the most to the USA. | The suggested line was included in the manuscript.  Lines 60-61: “Phytosanitary… … USA.” |
| **Materials and methods:** Lines 93 – 94 topographical conditions are not comparable to the national average; the most favorable conditions for avocado cultivation are in an altitude range between 1000 and 2300 meters above sea level. Won't this variability of conditions generate an error in your method? | The altitude value for the study region has been corrected, including only the altitude range of producing regions.  Lines 97-99: “Between… …sea level.” |
| In Figure 1, the level curves confuse with the flat areas, it seems that the flattest areas are to the northwest of the municipality. Remove the level curves or the physiographic legend. | Figure 1 was modified to avoid confusion. |
| Modeling of production systems: the conditions of your UPA are different from those of avocado crops in Michoacán and Jalisco. Consider this information if you are going to make national comparisons. In general, the methodology seems very solid | It was clarified that the system operates under rain-fed conditions to avoid confusion with other methods used nationwide.  Lines 137-138: “These… … rainfed.” |
| **Results:** line 268 – 269 Are the yield values average across the three states? There may be a lot of variability between production types in these three states. The results presented are precise and concrete. | The paragraph was revised to emphasize the differences in average avocado yield according to tree age.  Lines 273-277: “Plantations… … [20, 36].” |
| **Discussion:** lines 378 – 370 This data is relevant to your results. Can it be said that production in this municipality is not economically and socially profitable? When comparing data from other states or countries, it seems that the result is always different. Is this because the crop is relatively new in the area? | A new sentence was added to further elaborate on the profitability of avocado production.  Lines 392-394: “Developing… … profitable.” |
| Line 408 – 409 It is clear that protection for export producers is always more important, including plant health protection. | A sentence was added to address the comment.  Lines 456-457: “This… … [16].” |
| Line 442 -444 This data is relevant. It should be discussed further. | The idea was expanded to emphasize the impact of indirect taxes on small-scale avocado producers.  Lines 463-469: “These indirect… … mechanisms.” |
| Line 469 Could it be that avocado cultivation is not favorable for small farmers? Your discussion highlights that international and export trade is not viable or favorable for poor farmers, or at least Mexico's public policies do not favor them. | A sentence was included to highlight the importance of small-scale avocado farmers.  Line 494: “However… … fruit.” |
| **Reviewer 3:** |  |
| Please consider including the standard deviations for the data obtained from the 11 surveyed farms. This would enhance the reliability of the analysis. This could be presented in the Supplemental Data if space is limited in the main text. | A new table was included with weighted standard deviation based on technical coefficients.  Line 280: “Table 2…. …. coefficients.” |
| The resolution of the map provided is low, making the legend difficult to read. A higher-resolution version would be appreciated. | Figure 1 was modified for better understanding of the analyzed region. The Legend was simplified, and text resolution was improved.  Line 103 |
| The term "CNMPP" is used without a clear definition in the main text. Please provide an explanation. | The correct term is NPCI (Nominal Protection Coefficient for Marketable Inputs), and this abbreviation has been applied consistently across the manuscript. |
| While "NPCI" is defined in the Materials and Methods section, it does not appear to be reported in the Results. Please clarify or include the relevant results. | There was some confusion regarding the term CNMPP; it has been replaced with NPCI, and the corresponding paragraph has been added.  Line 347: “Nominal… … (NPCI). |
| Results – Yield (Line 265–): The fruit quality classification is said to follow reference [37], but the explanation is not sufficiently clear. Please provide more detail on how the classification was carried out. | The classification in accordance with Mexican regulations was expanded, and a table was added.  Line 291: “Table 3. Fruit… |
| Tables 2–4: The captions should include a brief explanation of the term “Divergences” for better reader understanding | The tables including the term “divergences” were removed and a figure was included as replacement.  A brief explanation of the term “divergence” is included in lines 180-182. |
| Tables 3–4: Some formatting issues are present in the tables. Please revise to ensure all contents are clearly readable. | The tables mentioned were removed. |
| Tables 2–4: Presenting the information for APU1 to APU3 in a single consolidated table may improve readability. Alternatively, showing the profitability by tree age using a time-series graph could help better illustrate the policy impact. | Tables 2, 3, and 4 were removed, and a new figure was added to summarize the results.  Line 302: “Figure 2. Profitability…”. |
| Table 5: Please revise the column header “Age” to “Age of Avocado Trees” for greater clarity. | The comment has been addressed. |
| Line 309: The reference to “Table 1, 2, and 3” should be corrected to “Table 2, 3, and 4.” | The tables mentioned were removed. |
| Line 309: The sentence “[The main outlays, constituting 43%, 48%, 55% of total costs respectively, were for fertilizers.]” is unclear. Which figure or table supports this claim? If the data are not presented, please consider including them. | A figure was added to support the argument.  Lines 322: “Figure 3. Marketable…” |
| Discussion: The paper attributes market failure primarily to low wages. However, could other factors also be contributing? A more nuanced discussion would strengthen the argument. | The discussion was expanded to include additional factors that may contribute to avocado market failures.  Lines 534-542: “Market failures… … [54].” |
| The authors mention that support for smallholder farmers is necessary. It would be helpful to suggest specific policy measures or practical approaches to make this recommendation more actionable. | A practical focus was incorporated into the recommendations.  Lines 516-519: “Addressing… …. Development.” |
