# Supplementary material for: Economic analysis of avocado production systems: Market failures and policy distortions
Source: PLoS One. 2025 Aug 25;20(8):e0330326. doi: 10.1371/journal.pone.0330326 (PMC12377620; doi:10.1371/journal.pone.0330326)
Supplement: Attachment — Submitted filename: PONE-D-24-45504R1 (Rebuttal letter 2).docx [file pone.0330326.s005.docx]

In response to the reviewers' feedback, the following changes were made to the original manuscript:

**Reviewer 1:**

| Line 131, “with ranges” should be replaced with “with age ranges”. | The correction has been addressed. |
| --- | --- |
| Line 280, you mentioned in Table 2 for APU2 (7-9), APU3 (10 to 14), in Table 3 for APU3 (7m10a14) and in Table 5 for APU2 (6-9), APU3 (>10), with respective age ranges, but they are not in line with what in materials and methods (Line 132-133, where APU1 (4-6 years), APU2 (8-9 years), APU3 (10-15 years). Their descriptions should be consistent throughout the paper. | The terminology was homogenized throughout the manuscript. |
| In Table 2 (Line 280), there is a misspelling, “tres”, which should be “trees”. | The correction has been addressed. |
| The names of Table 1, 2, and 3 are too short and not self-explained, which need a bit more words. | The title of tables 1, 2 and 3 were modified. |
| Figure 2 does not show any unit. | Units were included in figure 2. |
| Line 498, 542, the quotes (“”) are not necessary for the words “harms” and “distorting”. | The correction has been addressed. |
| You referred Table 6 in Lines, 341, 354, 459, 467, 375, but I could not find Table 6 anywhere. | The table that was referred was Table 5 not 6. |
| In Table 4, you need the unit (m or meter) in column 2. | The unit of measure for Table 4 was included. |
| In Figure 3, the APUs is described in title, but it was not included in figure, instead you mentioned 6m4a6, 6m7a9 and 7m10a14, for which I would suggest just to write APU1, APU2 and APU3 simply. | The legend in figure 3 was modified. |
